# Dematerialized participation challenges: Methods and practices for online focus groups

**DOI:** 10.3389/fsoc.2023.1145264

**Published:** 2023-04-06

**Authors:** Donatella Poliandri, Monica Perazzolo, Giuseppe Carmelo Pillera, Letizia Giampietro

**Affiliations:** INVALSI, National Institute of Evaluation of Education and Training System, Rome, Italy

**Keywords:** online data collection, online focus group protocol, methods in qualitative inquire, organizational setting, textual analysis, school principals, teachers

## Abstract

This study explores the limitations and benefits of different approaches to conducting online focus groups and illustrates an online focus group protocol used within the Value for Schools project in Italy. According to the project evaluation design, 13 online focus groups were organized, with the participation of 101 teachers and 37 school principals. The protocol setup, incorporation, and reorganization of the indications have been discussed in the literature, addressing the methodological and practical issues, such as the selection of participants and preliminary communication with them; the web conference platform (Zoom Business); timing, as well as access times and mode; the roles of the researchers involved (moderator, co-host technical assistant, co-host-observer, co-host-animator) and their integration spaces; technological support; and animation tools. The recording and transcription tools and subsequent analysis of the textual corpus are presented. Finally, the authors discuss the validation and reliability of online focus group protocols.

## 1. Introduction

In the digital era, new research methods use online technologies based on web applications and computer-mediated communication (Morgan and Lobe, 2011). In the field of social sciences, data collection is possible through different methodologies and tools in the online environment, such as web surveys, online qualitative interviews, virtual ethnographies, and online focus groups (OFG). While for some of these methodologies (questionnaires and interviews), the Internet is now replacing previous media such as mail and telephone, moving online is more problematic for others. In the preparation of OFGs, some scholars drew a continuum among methods that can be moved online with minimum adjustments while others needed to be deeply adapted to function in an online version. While some researchers note that collecting qualitative data online is limited by the lack of information about the physical environment and non-verbal behaviors, others have provided results that contradict such criticisms and show the same rigor in computer-mediated communication as in face-to-face communication (Morgan and Lobe, 2011).

The restrictive measures imposed by COVID-19 require that research strategies respond to changing needs. Online strategies should be considered “less as an adaptive compromise within pandemic restrictions” and more as an opportunity for innovative methodological paths (Keen et al., 2022, p. 2).

In this study, we describe, discuss, and validate a procedure related to 13 online synchronous focus groups, which were originally designed to be conducted in person but were moved online due to the COVID-19 pandemic. In the following section, we present a literature review on OFG, discussing its benefits and limits. Therefore, the study design, management protocol, step-by-step procedures, and technological tools are indicated. Then, we discuss the elements of method validation, including precision and accuracy. The results in terms of focus group validation, evaluation, limitations/benefits, and lessons learned are presented. Finally, conclusions are presented regarding how to capitalize on online video conferencing knowledge to further implement best practices for the benefit of similar studies in the future.

## 2. Online focus group

The focus group is a research method developed by Robert Merton and his colleagues. It serves to collect more in-depth qualitative data on participants' experiences, and it is a “research technique that collects data through group interaction on a topic determined by the researcher” (Morgan, 1997, p. 6). The focus group uses a group discussion on a specific topic or issue, guided by a moderator. The focus groups with 8–10 participants allow for richer and deeper information to be gathered because the participants may gain insights from the answers already provided by others (Agan et al., 2008; Krueger and Casey, 2014). According to the theories of symbolic interactionism, “all focus group participants simultaneously conceive their own role in relation to the roles that others play, finding the meanings of their actions in the reactions of others” (Morgan, 2012, p. 161). The interactions between participants are influenced by the group context and the processes of sharing and comparison with other participants. Therefore, social context cues are essential in establishing high-quality interactions in focus groups (Lobe, 2017, p. 236).

An OFG is a computer-mediated communication event (Albrecht et al., 1993; Lobe, 2017); it can be defined as “a selected group of individuals who have volunteered to participate in a moderated, structured, online discussion in order to explore a particular topic for the purpose of research” (Peacock et al., 2009, p. 119). The literature distinguishes between asynchronous focus groups, synchronous ones, and virtual world focus groups. In the asynchronous focus group, participants do not interact in real time. It requires minimal time to read and review questions. In synchronous focus groups, people participate in real time *via* instant messaging or videoconferencing (Williams et al., 2012; Tuttas, 2015). In focus groups in virtual worlds, Massively Multiplayer Online Games (MMOG), or avatar-based focus groups, participants interact in computer-simulated environments through personal avatars (Bartle, 2003; Houliez and Gamble, 2012). This method requires participants to be able to attend a specific platform and have sufficient expertise to engage with it.

According to Lathen and Laestadius (2021), OFGs can ensure full and equal participation for people who might otherwise face barriers. From a theoretical point of view, validity could be improved because participants can participate anywhere. It may also provide more opportunity to recruit an adequate and appropriate sample to add rigor to a study (Higginbottom, 2004; Morse, 2015), avoid selection bias, and optimize external validity (Kerr and Murthy, 2004; Bruüggen and Willems, 2009; Krueger and Casey, 2014; Wilkerson et al., 2014; Rupert et al., 2017; Daniels et al., 2019). OFGs make it possible to expand the audience of potential participants, recruiting them from remote places or in precarious health conditions, who might otherwise be excluded from the traditional recruitment process. This consideration is particularly important when researchers attempt to engage vulnerable populations to obtain adequate information on sensitive issues, such as opportunities for access to care (Dos Santos Marques et al., 2021). According to some scholars, virtual methods such as online focus groups can enhance studies in marginalized communities and, thus, mitigate the effects of social desirability evoked by unknown institutional contexts (Daniels et al., 2019). Sessions can be conducted over a variety of electronic devices (desktop, tablet, and mobile) with audio and text options available for those with insufficient bandwidth or technology.

Data collection through OFGs can be beneficial in terms of cost reduction (Joinson, 2005; Cater, 2011) and time investment (O'Connor and Madge, 2003; Jankowski and van Selm, 2005). OFGs can also bring together participants from different parts of the world, allowing for alternative time slots optimized for different time zones (Muttiah et al., 2016). From a technological point of view, the OFG offers advanced audio and video recording capabilities; it allows automatic transcription, including live captions. During the focus groups, different tools can be used to support discussions, such as instant messaging options or screen sharing, taking advantage of the support of virtual whiteboards/bulletin boards and being able to share images, files, etc. in real time. These tools expand the possibilities for supporting discussions by stimulating creativity and can remedy the lack of conventional facilitation supports for thematic focusing (e.g., blackboards). However, the use of virtual artifacts may be less natural than their physical equivalents, requiring the selection of user-friendly tools and staff with advanced tool management skills. According to the literature, it is necessary to carry out a preliminary setup/verification: indeed, any difficulty translates into the emotional detachment of the participants and, therefore, their further isolation. Based on these technological constraints, new and different responsibilities are identified for the people who support the moderator. In the literature, two or more support figures in addition to the moderator are often reported, both for the management of any technical problems and as support to the moderator to effectively lead the discussion (Wilkerson et al., 2014; Dos Santos Marques et al., 2021).

As for the limitations, first, the online focus groups are exposed to greater risks associated with dematerialized participation. Some scholars have argued that the absence or reduction of non-verbal or paralinguistic communicative elements reduced spontaneity, resulting in boredom, distraction, and stress generated by computer use, which can lead to a participation deficit, the latter aspect being particularly important when attempting to elicit further comments (Oringderff, 2004; Morgan and Lobe, 2011; Stewart and Shamdasani, 2017). Despite the possibility of reproducing an interactive situation, like a face-to-face one, some relational aspects are not guaranteed in the OFG, such as the use of all the senses directly involved, or some elements of the “pleasure of being there,” which is indispensable for generating trust and catalyzing “group energy”. Research in this area has shown, for example, that while participants in online meetings tend to provide shorter comments and offer brief hints of agreement during the discussion, in the case of face-to-face meetings, some participants tend to speak at length while others remain relatively silent (Bruüggen and Willems, 2009; Lijadi and van Schalkwyk, 2015). However, other research has shown that online focus groups are able to obtain an equivalent quality of data from participants (Underhill and Olmsted, 2003; Reid and Reid, 2005; Woodyatt et al., 2016; Abrams and Gaiser, 2017), and there are many similarities, including reliance on social cues and stereotypes based on gender, race, attractiveness, and age (Groom et al., 2009; Hoffman et al., 2012).

Second, OFGs can exclude communities and individuals without technical skills or access to appropriate devices and software (Namey et al., 2020). Participants may have various levels of digital skills, and even those who regularly use the Internet for other purposes may not have used video conferencing software. Participants unfamiliar with the technology may present additional challenges to the research team (Lobe and Morgan, 2021). In these cases, at the start of the focus group, it is advisable to train participants to use the relevant software. When setting up the focus group, researchers must consider how to correct socioeconomic, age-related, and individual disparities in the access to and the use of computers, the Internet, and video-calling technologies.

Third, there are also some methodological disadvantages. According to some scholars, the use of automatic transcription in virtual research may indicate that researchers become less familiar with their data than those who undertake manual transcription because deciphering interviews helps researchers absorb their content. Since researchers do not visit the contexts under investigation in person, they should explore the cultural context in alternative ways (Roberts et al., 2021). Additionally, participants in online focus groups are likely to be susceptible to distractions if they are in home environments: from childcare and pet care to work email notifications. Some participants also reported distraction due to seeing themselves on screen (Deakin and Wakefield, 2014). Additionally, video calls can tire participants faster (Epstein, 2020). It is also difficult to support remote participants if they experience emotional distress during interviews.

Finally, new technologies require updated ethical scrutiny (Salmons, 2016) in terms of consent, data confidentiality, stored focus group registrations, or automatic data transcriptions. While some scholars believe that most ethical concerns are the same as those in face-to-face research (Lobe et al., 2020), others have raised concerns over data confidentiality and cyber safety (Roberts et al., 2021).

## 3. Study design

In this study, we present and validate the protocol of 13 online synchronous focus groups, which were originally designed to be conducted in person but were moved online due to the COVID-19 pandemic. Synchronous OFGs allow for more fluid discussion, providing a closer approximation to traditional in-person focus groups (Tran et al., 2021). We considered both the target group's familiarity with the tools to be used and the habits of a category (teachers and school principals) in relation to the use of technologies. Indeed, during the pandemic period, due to the need to implement new distance learning strategies, teachers' familiarity with IT tools increased and greater preferences were noted for web platforms but not for instant messaging and group chat tools (Perazzolo, 2021; Zoja, 2022).

We adapted the salient aspects of the traditional focus group to the online versions, recognizing the importance of considering both methodological indications drawn from the traditional focus groups and the more recent online adaptations to properly combine and organize the elements. The challenge then is to be able to model, even in an online environment, conditions that support creativity within work paths that are necessarily more structured and tend to be less interactive and spontaneous than face-to-face meetings. The response to this challenge requires a careful preparatory organization, which is not limited to translating the work techniques used face to face but reflects in depth the ways in which communication is conveyed between facilitators and participants and between participants. The different phases of the focus group must be planned, all the proposed activities structured, and the tools able to support them identified, clarifying their usability, limits, strengths, and implications. The challenges, therefore, are regarding not only how to use technology to build an online focus group but also what methods of interaction and technological tools are most suitable for the research objectives of the focus group.

In the following paragraphs, we attempt to provide some answers to these questions by illustrating the building process of the OFGs within the Valu.E for Schools project *(VfS*). The *VfS* project has allowed the activation of three training paths—one for each macro-region of the country (northern, central, and southern)—with the double aim of strengthening the evaluation and improvement design skills of the teachers and school principals and of offering guidance to policymakers on networking, training, and support models in the field of school self-evaluation (Gomez Paloma et al., 2020).

The project involved 42 Italian schools, including 42 school principals and 400 teachers. Through the 13 OFGs carried out, a merit sample of project participants—principals and teachers—debated together the strengths and limitations of the training course and the medium-term progress of the project. The sample (recruited as explained in paragraph 3.1) consisted of 101 teachers and 37 school principals distributed in the 13 OFGs, as shown in **Table 2**. Of the teachers selected, 92 were women and 9 were men, with an average age of 52.4 years, an average career of 23 years, and an average seniority in the same school of 14 years. They were all permanent workers: nine working in a kindergarten school, 49 in a primary school, 41 in a lower secondary school, and two in administrative positions. Among the teachers, 95 of them held positions of responsibility within their own school and/or were members of the school self-evaluation teams. Among the school principals, 10 were men and 27 were women, with an average age of 54.7 years, an average career of 7 years, and an average seniority in the same school of almost 5 years.

The spatial-temporal context of OFGs was the pandemic, specifically in the spring of 2021.

The protocol identified some implementation indications, such as group capacity, the web conference platform used, the communication model for the participants (cover letters, emails with the link), the length of the meetings, the roles of the researchers involved (moderator or host and co-hosts) and their integration spaces, technological support, and animation tools. Among these indications, the following played an important role: the multimedia presentations of the stimuli; the chat (with the possibility of posting comments relating to interventions and of intervening despite video connection problems); the private chat between researchers; the final word cloud (generated by the real-time automatic transcription of the speeches); and the final group photo.

### 3.1. Step-by-step OFG protocol

We detailed the OFG protocol using a three-step approach: Step 1—Online focus group design and planning, Step 2—Online focus group implementation, and Step 3—Online focus group postproduction and analysis.

#### 3.1.1. Step 1—Online focus group planning

The first step included four main activities: the definition of the aims of the focus group based on the research design; the recruitment of participants; the preparation of the technological tools; and an online focus group pilot test.

We identified four research questions and we modulated the focus group stimuli in relation to the professional roles of the participants. In the meantime, we selected the online platform most suitable for our purposes, we reflected on the technological and animation tools to be used during the meetings and drafted a protocol as a supporting tool and outline for work development (see below, par. 3.4). Lastly, we planned the communication procedures with participants.

The participants in the focus group study were selected based on their participation in *VfS* training. Recruitment was done *via* an email invitation to the principals and teachers. Names, email addresses, and professional roles of the participants were collected. Other data were collected from the surveys that the participants had filled out in the previous months. The confirmed participants of the OFGs were emailed the online meeting details, including the date, time, and video conferencing platform login instructions.

Then, a pilot test was conducted with the moderator, co-hosts, and two volunteers as participants. The OFG pilot tested the suitability of the platform and other technological tools along with the format and content of the session; it was useful to become familiar with all the features of the video conferencing platform and to practice the staff roles (moderator, co-host animator, co-host observer, co-host assistant).

The OFG planning step lasted 2 months, and it was carried out by six researchers to address the methodological, technological, and contextual aspects of the method.

#### 3.1.2. Step 2—Online focus group implementation

The focus groups were scheduled in the afternoon sessions, starting at 4.30 pm, for a duration of 90 min. On the morning of each scheduled OFG, an email reminded participants of the meeting URL. On the scheduled meeting date/time, participants clicked on the URL to access the online meeting. Participants were sent an alert 15 min before the start of the focus group (4.15 pm). Arriving 15 min early ensured participants were logged on successfully and could troubleshoot any technical issues. According to the literature, this is a good practice to increase the likelihood of starting and ending on time, thereby demonstrating respect for participants' time (Tuttas, 2015, p. 126).

**Figure 1:**
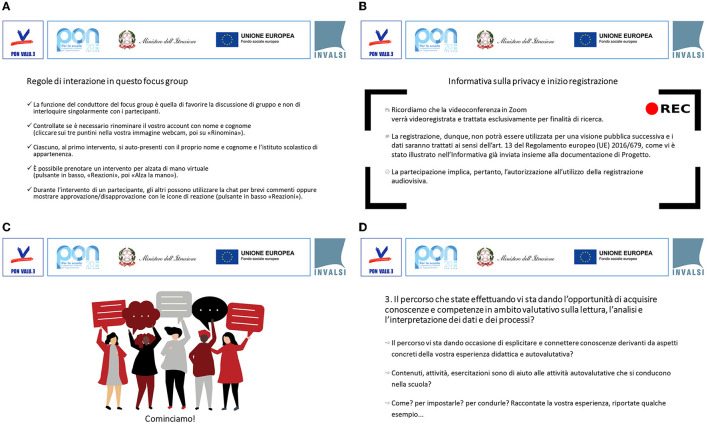
Examples of slides shared on-screen during the OFGs: to illustrate the interaction rules **(A)** and the privacy policy and to signal the start of video recording **(B)**, to start the discussion **(C)**, and to launch stimuli and related sub-questions **(D)**.

Upon starting the focus group, an introductory slide was shared containing the name of the session. The moderator began the session by introducing the aims of the focus group and provided a brief reference to the rules of interaction during the meeting (Figure 1A). During this phase, participants were informed that the focus group would be recorded for transcription purposes (Figure 1B). The staff had their camera on all the time, and the moderator invited everyone to join the meeting with video, even though it was not mandatory. Almost all the participants kept the video active after the start (Figure 1C). Within the focus group script, four questions were provided along with relative comments that were used to guide/ensure comparable conversation among the various OFGs (Figure 1D). The moderator maintained an active role by asking questions, clarifying the answers, and ensuring that every participant was given opportunities to participate. The final part of the focus group session included a summary of the issues that had emerged, exemplified through a word cloud (see below, paragraph 3.4.2). Before the final goodbyes and thanks, a screenshot of the videos of all the participants (which counts as a “souvenir photo”) was presented. The representative keyword of the focus group, from the participant's point of view, was written on each participant's photo (see below, par. 3.4.3).

Following each focus group session, the staff held a debriefing discussion to identify aspects of the protocol that went according to plan and which aspects could be improved.

The OFG implementation step lasted 2 months.

#### 3.1.3. Step 3—Online focus group postproduction and analysis

In the third step, the deliberations of focus groups were stored on a server and protected by the research institution's data processing rules. The OFGs were then transcribed, and a quality check of the transcript was subsequently performed. The transcribed texts were prepared for analysis using computer analysis software.

The transcript quality control step lasted 1 week and was carried out by three researchers.

The analysis step is still ongoing and it is being carried out by five researchers. Multiple in-depth paths are possible. In Table 1, the technical equipment used is summarized. The software related to database building and data analysis (Cabolo, QDA Miner) is discussed below in paragraph 3.6.

**Table 1:** Software used and their purposes.

**Software**	**Purposes**
Microsoft 365 suite (Word, Excel, PowerPoint)	Drafting the cover letter/invitation to OFG and participants' data collection form, manual review of automatic transcriptions (Word); composing the lists of participants and building the database (Excel); creating a technical guide for the staff and multimedia presentation to be used during the OFGs (PowerPoint).
Zoom	Videoconferencing tool.
Bitly	Creation of a shorten, customized, and human-readable invitation link to every Zoom meeting.
Whatsapp (group)	Parallel/external communication tool reserved for staff.
Google Web Speech API and Dictation.io	Automatic real-time transcriptions of the meetings.
Voyant Tools	Filtering the automatic transcriptions of meetings by stop-word list and generation of word clouds.
Cabolo	Accurate automatic transcription of each meeting from the audio-visual recording.
QDA Miner	Text coding and analysis.

### 3.2. OFG management

In running the OFG, the areas that most influence its success and require greater attention include (a) the introduction of technological tools and their management by the participants, and (b) the arrangement of how good organization of the discussion among the participants can be achieved through a proper setting (Stewart and Shamdasani, 2017).

In the literature, the need to provide one or two support figures in addition to the moderator is reported, both for addressing any technical problems and as support to the moderator in the function of good supervision of the focus group (Wilkerson et al., 2014; Dos Santos Marques et al., 2021).

The moderator's role in facilitating the virtual focus group is like the role of leading in-person focus groups. The moderator sets the context, guides the discussion, and engages participants in an interactive conversation. The moderator also sets the tone for the conversation, allowing all participants to feel comfortable and engaged. However, the interaction takes place online, on the platform, and it is conditioned by technological characteristics and constraints; therefore, new and different responsibilities are identified for the people who support the moderator (Wilkerson et al., 2014; Dos Santos Marques et al., 2021).

Sometimes the moderator might not only have to manage multiple functions at the same time, responding to more than one intervention but also have to pay attention to interactions between participants, attempting to facilitate the flow of online conversations through the technological environment selected (Lijadi and van Schalkwyk, 2015). Managing an online participatory process can be quite complex because there is a greater risk of losing participants' attention. There may be background noise, distractions, and situations such as participants entering late and participants having problems with their connection, video, or audio, even mid-session. At the same time, some constraints due to the lack of physical proximity that may affect the conduct of online groups, e.g., absence, or reduction of non-verbal or paralinguistic communicative elements, boredom, and computer stress (Morgan and Lobe, 2011) should be managed to ensure effective interaction among participants, communicative quality of verbal exchanges, and fluidity of interactions. Furthermore, non-verbal or body language must be taken into account because, in light of these signals, the moderator should evaluate the answers as well as adapt their own behavior in response to the signals received from the group. Participant engagement is essential to the success of a focus group, and it is important that the moderator remains engaged to demonstrate that discussions are being followed up and taken on board. Participants can abandon or limit their effective participation if they are not involved, if they do not perceive the active role of the moderator, or if the environment created is not stimulating and pleasant (Terrell, 2011; Murgado-Armenteros et al., 2012; Stewart and Shamdasani, 2017).

OFG co-hosts must generally provide logistical support: they admit and organize participants in the waiting room, manage late arrivals, and help participants with technical problems during the session. Moreover, they can monitor the development of the ongoing session, observing which participants have contributed and which have not (Stewart and Shamdasani, 2017), checking and noting if there is any participant who has not yet spoken, and reporting this information to the moderator.

In our study, OFG management was carried out by four researchers; their roles were organized as follows.

- *Moderator*: The moderator moderates the discussion by paying attention to the participants and the indications of the staff. The moderator should therefore be very skilled in controlling the group, guiding the discussion, introducing, returning to questions, sharing summaries, providing feedback, guiding participants' interventions, and making sure that the conversation flows as smoothly as possible.
- *Co-host assistant*: The co-host assistant supports the technical management of the meeting, especially in the starting phase (supports the management of late arrivals), and shows the slides with the presentation of the event, the working group, the stimuli, etc.
- *Co-host animator*: The co-host animator manages the technical aspects of the meeting (setting up the environment, videoconference tools and materials, admission of participants) and technical problems faced by the participants, helps in the management of work tools such as shared blackboards and concept maps, and uses an automatic real-time transcription of the web conference to process one or more word clouds, etc.
- *Co-host observer*: The co-host observer keeps track of chat interventions and focuses on non-participating observation of group dynamics by privately and briefly sharing support information in real time with the moderator for the management of the discussion (see paragraph 3.4.1).

### 3.3. Ethical consideration and researchers' positionality

Regarding the ethical aspects, participants were informed of the OFG purpose by email invitation, and they were informed that the focus group would be recorded for transcription purposes. Participants were reassured about privacy and data procedure processing. All participants signed the informed consent provided for participation; all consent forms were collected and stored. The consent form informed participants about their right to withdraw from the study at any time.

According to Jacobson and Mustafa (2019), in qualitative research, it is important to map the social identities of researchers to support a better understanding of the data relations and account for them in a responsible and respectful way. Keen et al. (2022) suggests that researcher positionality in virtual research is based on the same considerations as in-person research (e.g., Salmons, 2016). In our virtual study, we believed that our outsider positionality—no members of the research staff were teachers or principals—required intentional actions to deeply understand our study context. Meanwhile, the research staff was part of the institution that financed the training course on which the participants were asked questions. For this reason, we carefully considered our positionality in relation to the participants' freedom to speak and express themselves, and we recruited an OFG moderator from outside our institution with a background as a school principal to support the process, starting from the definition of the stimuli.

### 3.4. Materials and equipment

#### 3.4.1. The videoconferencing system

Regarding the technical solutions to implement OFGs, it should be remembered that the choice of a particular videoconferencing system among the many possibilities, the activation of additional features, and/or the use of external applications to expand its functions must be subjected to careful evaluations. Electronic video-communication tools and their specific functions or add-ons (chat, file-sharing, blackboards, billboards, bulletin boards, surveys, etc.,) can certainly make up for the lack of conventional physical media for organizing and facilitating meetings and create a good degree of social presence, even in an online environment (Stewart and Shamdasani, 2017). Moreover, depending on the objectives of the focus group, they can expand the possibilities to support the discussion and stimulate creativity and collaboration among the participants (UXalliance, 2020). Nonetheless, the interaction with and through virtual interfaces presents a lower degree of naturalness than that based on similar physical artifacts in face-to-face meetings. Thus, any difficulty in electronic communication would result in an emotional and cognitive detachment of the participants and, therefore, in their further isolation (Murgado-Armenteros et al., 2012; Daniels et al., 2019). For these reasons, it appears necessary

- to consider the levels of digital literacy among the audience and the potential impacts of a digital divide;
- to select tools that are as user-friendly as possible;
- for the staff to possess or develop advanced expertise in the chosen virtual interface and electronic tools;
- to set up, fine-tune, and test all the tools and their settings in advance.

**Figure 2:**
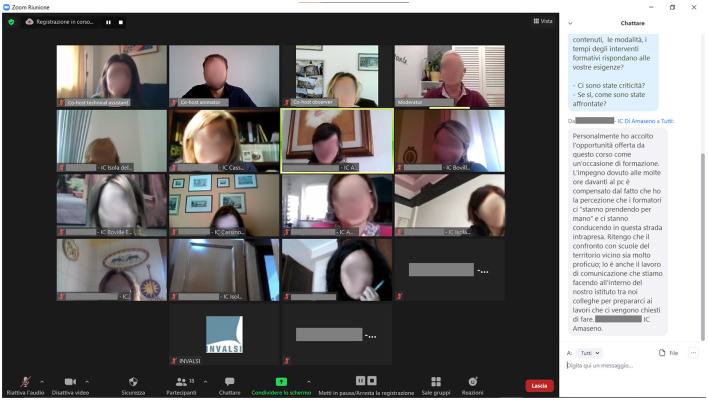
Example of a screenshot with a chat window that illustrates some interface setups of Zoom.

Considering these premises and the purposes of our OFGs, we opted for maintaining a simple and intuitive digital architecture, focusing simply on the videoconferencing system without external tools, except for a WhatsApp group, limited to internal staff communications (e.g., to suggest that the moderator solicit contributions from participants who have not contributed or that he explores a particular theme).^1^ After careful consideration (Wilkerson et al., 2014; Lobe et al., 2020)^2^, and much like several other experiences reported in literature (Kite and Phongsavan, 2017; Lobe, 2017; Matthews et al., 2018; Archibald et al., 2019; Daniels et al., 2019; Dos Santos Marques et al., 2021), our choice fell on the well-known Zoom platform (Figure 2), for the following main reasons: (a) our research institute already had a subscription to the “Business” version, which allows unlimited meeting time and was equipped with any additional functionality deemed necessary to support the meetings (integrated chat, shared whiteboard, on-screen document sharing, etc.,), as well as the possibility of advanced configuration of the meeting and saving both the audio-visual flow of each OFG and its complete chat log; (b) although dedicated software (client) that can easily be downloaded and installed on a variety of devices and operating systems is available, this is not necessarily required of participants when using Zoom, who can easily access the meeting *via* a web-browser too; c) the target audience was highly familiar with this videoconferencing system, which was used extensively in schools during the pandemic period.

Among the measures taken in the Zoom platform settings:

- choice of the “meeting” (not “webinar”) mode because the focus group is a highly interactive event;
- setting up the meeting video-recording feature using the cloud space provided by Zoom's “Business” subscription;
- preparation of a “waiting room” with customized text and logo;
- access to each meeting using a specific ID and password or through a specific pre-coded link generated by Zoom and forwarded to the participants in an abbreviated, human-readable, and customized way, through the free service offered by Bitly^3^;
- accreditation of the accounts from which the three co-hosts were connected as “hosts” of the meeting, thus endowed with special privileges such as the admission of other participants and screen sharing;
- manual admission of participants from the waiting room to the meeting room by the co-hosts;
- participants' access to the meeting room with muted microphones;
- activation of the integrated chat feature, to report verbatim on it, from time to time, the stimulus, and sub-questions of the corresponding discussion phase (after they had also been presented through shared-screen slides) and, for participants, to not only make written comments on others' interventions and send technical support requests but also to join the discussion if there were connection problems^4^;
- activation of the instant survey feature, tested “on the fly” only in some OFGs, to support some moderators' extemporaneous requests for specifications^5^;
- activation of the multimedia whiteboard, used exclusively by the co-host animator, as specified below, to support the back-talk phase.

Several of the previous settings can be saved in a template so that they can be recalled immediately for the preparation of a subsequent meeting. Overall, Zoom (with a Business license) proved to be an excellent choice, both in programming and in managing the OFGs. Apart from a few rare slowdowns when sharing the screen, there were never any hiccups or problems attributable to the platform or difficulties in its use by the staff or participants.

#### 3.4.2. The word clouds

**Figure 3:**
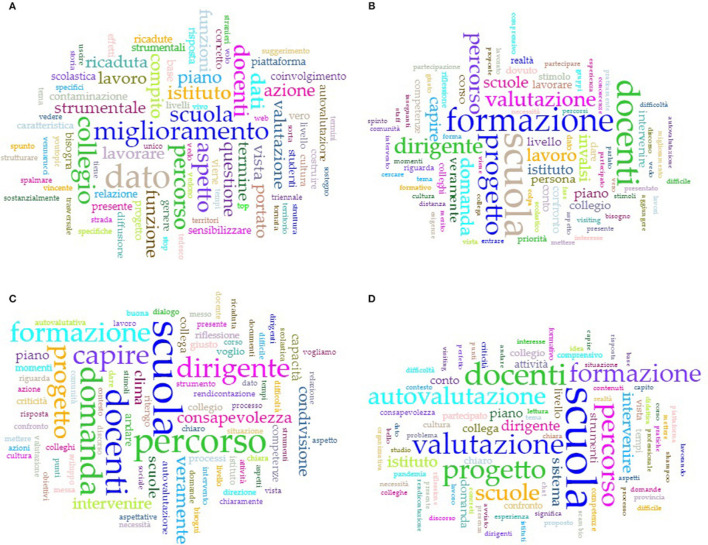
Word clouds of the school principals' OFGs: North **(A)**, Center **(B)**, South **(C)** Italy, and recovery session **(D)**.

In addition to supervising and supporting the meeting from a technical point of view, the co-host animator had the task of implementing the technical procedures for preparing a word cloud as a summary representation of the discussion.^6^ This was mounted on a slide and shared on-screen in the final phase of the OFG, with the aim of offering a final synthesis and supporting the moderator's back-talk (Figure 3).

To generate the word cloud, the co-host animator, after appropriately configuring the audio settings of their computer, automatically transcribed the audio stream of the meeting in real time, thanks to the free Web Speech API offered by Google^7^, as the analogous Zoom function is currently supported only for the English language. However, we recommend using this solution in conjunction with other free online applications that exploit its potential, since the dictation is interrupted whenever the window is dropped or minimized if you use the Web Speech API within its “natural” web application, i.e., Google Docs. Among the various online tools based on Web Speech API, tested with very similar results, we opted for Dictation.io.^8^ Web Speech API plus Dictation.io, used on the Google Chrome browser, have demonstrated an acceptable level of accuracy for Italian, provided that the audio stream is of good quality and volume.

**Figure 4:**
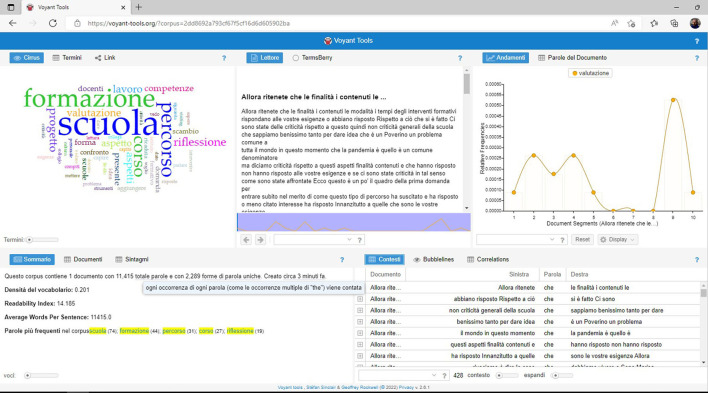
The work environment of Voyant Tools, a web-based text data mining suite used during the OFGs.

Once the transcript has been obtained and copied or saved in a text file, there are several possible solutions for the generation of word clouds, even free and web-based ones.^9^ One of the best solutions used in our case was the one offered within Voyant Tools^10^, an online text data mining suite (Figure 4) that presents, among many features, the possibility of quickly uploading and using a stop-word list to filter the corpus.^11^

#### 3.4.3. Souvenir photo with the keywords

**Figure 5:**
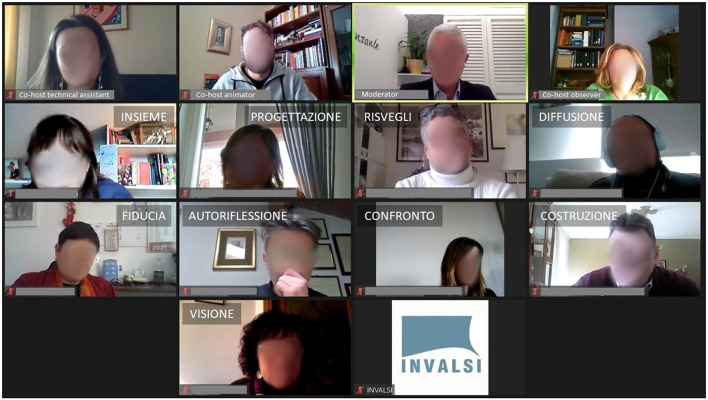
School principals' group photo with participants' keywords (macro-region: Center of Italy). From **top left** to **bottom right:** together, planning/design, awakenings, diffusion, trust, self-reflection, confrontation, construction, vision.

In the closing phase of each OFG, the co-host assistant chose one of the best screenshots taken during the discussion (paying attention/taking care that all faces were clearly visible and did not show grimaces, closed eyes, etc.), mounted it on a slide, and projected it on the shared screen as the group photo. At the same time, the moderator invited each participant, in turn, to summarize what the meeting had represented for/meant to them in one word, which the observer/technical assistant noted on the slide with the group photo, next to the portrait of the corresponding person (Figure 5). The slide could be sent to the participants as a souvenir photo.

### 3.5. OFGs evaluation form

To enhance transparency in our overall empirical study (Daniels et al., 2019), the OFG staff, at the end of each focus group meeting, documented their reflections in a specially developed and structured analysis form. The form was organized into four sections which reproduced the order of the presentation of the stimuli during the focus groups; in the last part, other evaluation notes on the conduct of each meeting were included, such as information on each focus group, including timing and any events during the group (e.g., technical problems), observations on the atmosphere of the meeting, and elements relating to participation and management. In this way, we noted, through progressive categorization, the strengths and differences highlighted by the participants with respect to the set of stimuli (Poliandri et al., 2022). These forms facilitated the first discussion among the research staff and the evaluation of the effectiveness of the methods implemented to collect data (Daniels et al., 2019). They allow us to reflect on the factors that facilitated the behavior of the group and to evaluate whether the adopted protocol worked out well.

According to the evidence collected, all OFGs took place in an atmosphere of great serenity, cordiality, and a spirit of discussion. All or almost all the participants showed pleasure in participating and willingly engaged in reflection, and they became progressively more relaxed and involved. Participants rarely talked over each other, and very rarely did the moderator have to intervene to stop someone's talkativeness. The moderator was able to create a relaxed atmosphere and put everyone at ease, also repeatedly emphasizing the usefulness of dialogical moments such as these. Sometimes it was necessary to solicit the interventions of the participants, but very little was enough for the moderator to set in motion a correct debate and to ensure that all participants expressed their opinions.

### 3.6. Data analysis protocol

#### 3.6.1. Transcription and database building

As research staff, we carried out corpus preparation work consisting of the following steps: automatic transcription (I), manual review of transcripts (II), and database building (III).

I. The audio recordings of the OFGs were automatically transcribed using a special online service called Cabolo.^12^
II. The text files containing the automatic transcripts were manually checked and deposited on a server with shared access by staff.^13^ The review was conducted by coming back to video recordings to
  a. correctly attribute each intervention to a speaker, identifying them by name and surname,
  b. fix punctuation, and
  c. correct semantic errors in the interpretation of the transcription software.

III. The revised transcripts were transferred to an MS Excel spreadsheet. The database thus built (henceforth DB) was structured in such a way that each row (a record) corresponds to a speaker's intervention, defined as a “fragment”, and endowed with a univocal, sequential, and *speaking* code (“unique fragment ID”), acknowledging that, with this code, the fragment can be identified at any time and possibly relocated according to the specific meeting to which it belongs and to the order of the interventions within it. The other variables, that populate the DB columns, have been variously obtained (preparatory materials, transcripts themselves, and satisfaction questionnaires administered to the entire sample of participants at the training courses).^14^

#### 3.6.2. Textual content coding and analysis

We set up the analysis of the OFGs according to a phenomenological-interpretative perspective (Moustakas, 1994; Merriam, 1998), proceeding using a qualitative approach to the textual content analysis (Losito, 2002; Mayring, 2014).^15^ Considering what the literature in the field proposes (Fereday and Muir-Cochrane, 2006; Adu, 2019), an abductive text coding methodology was developed, which combined

I. an inductive/bottom-up approach (first-level specific descriptive codes, emerging from the reading of the text) and
II. a deductive/top-down approach (second-level interpretative categories, grouping codes thematically related).

The inductive work of code construction and attribution was carried out by three researchers, as coders, and a coordinator. They worked exclusively on participants' fragments (920, stripping them of any final greetings from each meeting), while the moderator's interventions were used only for better understanding and contextualizing the emerging meanings. This research group, through a series of recursive steps operated on some focus groups used as tests (independent exploration and coding, comparison, construction of a code-set with label and a definition for each code, re-coding, analysis of inter-coders agreement, re-definition of the code-set, independent re-coding, etc.), came to an agreement on a definitive code-set that was used to independently code all other focus groups.

The sentence—understood as a single clause/proposition or a group of them between one full stop and another—was taken as the minimum unit of analysis for the attribution of a code. However, since punctuation marks were, to a certain extent, arbitrarily inserted (during the automatic transcription phase and subsequent manual revision, interpreting speakers' pauses and changes of speech), it became necessary to define certain rules to deal with coding in particular situations.^16^

**Figure 6:**
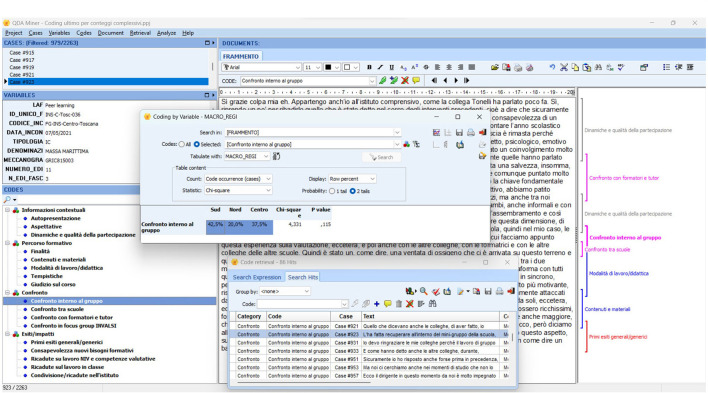
Example of the QDA Miner working environment for coding and analyzing content.

The entire coding process outlined above as well as the main subsequent analyses were computer-aided, feeding the MS Excel DB (see above, par. 3.6.1) to QDA Miner (Figure 6).^17^

Starting from the coding work, we developed several in-depth studies, focusing on individual codes or categories to clarify participants' expectations and elements of satisfaction and dissatisfaction, to study the processes of collaboration and exchange of experiences, examine the various types of training outcomes, and offer information on which topics and which training methodologies work according to specific target group characteristics. These studies used QDA-Miner mainly to apply techniques for analyzing co-variations/correlations (contingency tables, chi-square, Spearman's Rho, Pearson's R) between the coded cases and the background variables presented above in par. 3.6.1.

## 4. Discussion

### 4.1. Validity and reliability

From the early stages of planning research, it is useful to ask questions regarding how much the techniques identified to be used in the survey can provide valid and reliable data (Silverman and Gobo, 2002). This resulted in the need to explain the procedures used to ensure both the reliability of the technique used for our research and the validity of the conclusions. For this reason, we developed a protocol for OFGs that already facilitates the evaluation of the effectiveness of the method we used and, therefore, its validation.

To evaluate the effectiveness of the OFG protocol, we used Krueger and Casey's (2014) criteria for the constituent components of a proper focus group as a measure. The six key characteristics identified by Krueger and Casey were: (1) focus groups involve people; (2) focus groups are conducted in series; (3) participants are reasonably homogeneous and unfamiliar with each other; (4) focus groups are ways of collecting data; (5) focus groups make use of qualitative data; and (6) focus groups have focused discussion. According to Turney and Pocknee (2005), these criteria were in turn evaluated in relation to the online focus groups we conducted, clarifying the elements supporting the validity and reliability of the method (see below, paragraph 4.2).

Although the problem of validity and reliability is crucial in qualitative research as in any other type of empirical study, it is an often-overlooked problem (Lucidi et al., 2008). Similarly, the need to generalize the descriptions or explanations that qualitative research offers of a certain phenomenon is neglected (Bryman and Burgess, 1999). According to the classification of Kirk and Miller (1986), as far as validity is concerned, we can summarize that, in this study, we refer in general terms to validity as (a) a semantic correspondence of the results with the context from which the data are taken (*semantic validity*); (b) a concordance of what is highlighted through the use of different tools (*instrumental validity*); and (c) a method of reasoning based on criticism and continuous questioning of the conclusions reached (*theoretical validity*), if they can be valid outside the contingent condition in which they were obtained (*theoretical generalization* vs. *distributive generalization*; Hammersley, 1992). As far as reliability is concerned, we considered it as the replicability of procedures, distinguishing between internal and external reliabilities (Seale, 1999).

Within our OFG protocol, the semantic validity was evaluated through the so-called validation of the respondent (Lincoln and Guba, 1985), both through the first results emerging from the focus groups and the main research findings, to evaluate the degree of coherence attributed by the subjects studied to the meanings of the interpretive categories identified by the researchers. Our protocol allows for (1) collecting direct feedback from focus group participants through the souvenir photo tool where some keywords are highlighted; (2) collecting indirect feedback from the participants through tools such as the researchers' analysis form highlighting the climatic aspects; the word cloud (which represents an indirect measure of attention to the stimuli presented by the researchers and the return of words that have taken up the overall script of the proposed stimuli); and some quantitative measures of participation in focus groups, such as average speeches per participant and speeches per participant in 60 min (see Table 2); (3) carrying out computerized textual analysis of the transcripts made automatically in the focus groups, which highlight specific participation references; and (4) organizing seminars to present findings to evaluate the interpretative categories created by the researchers. In our opinion, the use of technology enhanced the aspects of semantic validity, offering a wide range of direct and indirect feedback for semantic validation.

**Table 2:** Characteristics of focus groups, participants, and metrics of textual database.

	**Length (min.)**	**Schools**	**Participants**	**Speeches**	**Average speeches per participant**	**and STD DEV**	**Speeches per participant in 60 min**.	**Token**	**Type**	**Token/min**
North-Emilia-Romagna, Teachers	109	6	14	78	5.57	3.59	3.07	15,289	2,492	17.77
North-Lombardia, Teachers	90	4	7	61	8.71	5.96	5.81	12,560	2,214	15.86
North-Piemonte, Teachers	96	3	10	84	8.4	4.93	5.25	13,245	2,117	15.34
Center-Lazio, Teachers	110	5	13	89	6.85	4.22	3.73	15,592	2,490	17.57
Center-Toscana, Teachers	106	3	9	80	8.89	3.06	5.03	14,411	2,273	16.72
Center-Umbria, Teachers	103	4	10	73	7.3	3.59	4.25	15,342	2,489	16.71
South-Campania, Teachers	116	5	18	97	5.39	1.91	2.79	16,007	2,565	18.59
South-Puglia, Teachers	117	4	10	111	11.1	6.47	5.69	17,250	2,603	17.66
South-Sardegna, Teachers	103	4	10	109	10.9	5.72	6.35	14,270	2,257	16.29
Nord-School Principals	105	9	9	70	7.78	3.19	4.44	14,167	2,244	16.63
Center School Principals	102	9	9	70	7.78	3.77	4.58	14,567	2,436	17.06
South-School Principals	116	9	9	101	11.22	4.02	5.80	15,366	2,545	19.20
All area-School Principals	99	10	10	116	11.6	7.2	7.03	14,331	2,439	16.85
Total	1,372		138	1,139				192,397	31,164	
Average	105.54	5.77	10.62	87.62	8.58		4.91	14,800	2,397	17.10

As far as instrumental validity is concerned, we can consider it the degree of agreement established through triangulation between the results of different data collection and/or analysis tools with respect to the same object of study. The basic idea is that, if some aspects of the phenomena and/or relationships between the phenomena are highlighted with more than one method, these are valid. In our protocol, we constantly verified the concordance between the issues that emerged in the focus groups and those investigated by design in the online survey, highlighting the consistency between the two.

In our study, with the aim of identifying coherent relationships between the initial hypotheses or those developed during the study and the results obtained to strengthen the theoretical validity, the definition of the sample of participants was a reasoned choice. Cases were selected based on their status in one or more properties, exemplifying the research topic. Participants were selected with the aim of clarifying some aspects of the general theory underlying the research. Therefore, the OFG sampling we conducted is theoretically significant. We attributed crucial relevance to the choice of cases (Flyvberg, 2006). The type of generalization that we evaluated in our protocol can also be referred to as “transferability” (Guba and Lincoln, 1989) or analytic generalization (Yin, 2000), i.e., beyond the contingent differences of context, the possibility of identifying a set of statements that can shed light on the behaviors, values, beliefs, typical of cultures of similar life forms (organizations) (Ricolfi, 1998). While on the one hand, the organization of online focus groups may have facilitated participation given the possibility of participating without moving, on the other hand, we do not know if some subjects may have chosen not to participate due to the digital device. If the theoretical validity manifests itself in the attempt to verify the hypotheses that are progressively formulated based on the empirical relationships traceable in the data (Silverman and Gobo, 2002) and the provisional analytical scheme must always be compared, even with the negative (even absent) or deviant cases, due to also not being able to determine a priori, the non-participations can constitute a threat to this type of validity. Surely this is an aspect that will have to be improved in the future to strengthen the theoretical validity of OFGs.

The extensive use of technologies, both for the management and animation of OFGs and for the construction of the empirical base and data analysis, greatly increases the reliability of the technique, as this essentially depends on the explicit description of the observational procedures and the transparency of the analysis process. Most innovations that tend to be predominantly technological (audio-video recordings, automatic transcription software that allows text to be structured and therefore information to be inserted into structures and transformed into data, also for carrying out quantitative analyses) can allow for an increase in the degree of inspection/explorability of the empirical base. Furthermore, technologies facilitate greater recurrence by switching back and forth between theory, collection, and data analysis, which is one of the characteristics of qualitative research, helping to make it more rigorous (Ricolfi, 1998). In our study, we defined the protocol that we present in this study to exhaustively explain all the procedures used and systematically organize the data collected. The use of technology has made it possible to strengthen and enhance the external reliability of the focus groups we have conducted, increasing the likelihood that all the needed information has been provided so that other researchers can replicate the procedures of our study, not necessarily to obtain the same results but to understand differences.

Furthermore, the possibility of having an immediate transcription of the texts available has increased our ability to conduct computer-aided textual analysis, being able to operate a continuous return to the empirical base constituted by all the audio-video material, increasing the degree of stability, reproducibility, and accuracy of the results achieved (Lucidi et al., 2008).

### 4.2. OFG evaluation effectiveness

#### 4.2.1. Focus groups involve people

As shown in Table 2, 37 school principals (out of 42 eligible) took part in four online focus groups, and 101 teachers (of 126 eligible) took part in nine online focus groups, one for each region involved in the project. An average of 10.7 people took part in each focus group: from nine to ten in those for school principals; from seven to 18 in those dedicated to teachers. A higher number of participants took part in OFG for the Campania teachers, because during the admission of the participants to the session, the staff was hesitant to exclude participants who had not confirmed.

On average, across all OFGs, there were 8.58 speeches per participant (Table 2).

The participation experience and feedback on the focus group by participants were categorized into a specific code called “focus group internal debate”, through computer-aided textual analysis (QDA-Miner software). This analysis found that the participants reported enjoying the online focus groups. For the participants OFGs were (1) a way to connect people who attended the *VfS* training; (2) an opportunity for comparisons between different schools; (3) an opportunity for further reflection and meta-cognition on the training experience; (4) a learning phase; and (5) a synthesis phase of the meanings attributed to the training by the beneficiaries and the research group. Participants recognized the moderator's role of true listening and effective synthesis. Several times, the moderator and co-hosts received congratulations for the organization and appreciation for the educational value attributed to the implementation of the focus group itself. These findings are related to studies where OFG participants reported enjoying having their voices heard and connecting with others during the pandemic and finding online focus groups to be supportive (Lathen and Laestadius, 2021; Keen et al., 2022).

Finally, in the souvenir photo, participants summarized in one word what the meeting meant to them: out of a total of 135 occurrences, 87 words were used, all with positive connotations, such as orientation, sharing, motivation, course, pleasant, innovation, change, imagination, training, trust, reflection, debate, surprise, collaboration, inclusiveness, introspection, relationship, together, planning/design, awakenings, diffusion, production, and vision. The most frequent words highlighted were sharing (14), debate (13), reflection (8), and productive (4).

#### 4.2.2. Focus groups are conducted in series

In this study, 13 OFGs were carried out, between the end of March 2020 and May 2020; four OFGs involved school principals, one OFG for each macro area (northern, central, and southern Italy) plus one for recovery. Nine OFGs were held for teachers. Each focus group lasted on average 105 min. Overall, the OFGs consisted of a total of 1,339 min of video recordings (see Table 2). The same script and outline for each OFG steered the debate around the research topic. According to Krueger (1994), it is necessary to run multiple groups with similar participants to optimize the data collection and group trends. The adoption of a protocol for the management of focus groups facilitated the possibility of giving uniformity to the various sessions of focus groups, which were held on different days and with different participants.

#### 4.2.3. Participants are reasonably homogenous and unfamiliar with each other

In this study, each focus group involved groups of homogeneous participants. We organized two kinds of OFG based on the homogeneity of the role and course attended by participants: one targeting school principals grouped by macro-regions and the other, teachers grouped by region. At the same time, the participants did not know each other. Indeed, as Krueger (1994) says, it is necessary to recruit participants with shared interests, because it allows them to focus the debate and to express controversial or private points of view. However, on the contrary, Krueger and Casey (2014) argued that familiarity tends to inhibit debate.

#### 4.2.4. Focus groups are methods of data collection

In this study, the OFG was used with the specific goal of collecting data on the strengths and limitations of the *VfS* training course and the medium-term trend of the project. Video recordings and text transcription made it possible to record participants' responses accurately and automatically. The revised transcripts were transferred to an MS Excel spreadsheet, in which each row (a record) corresponded to an intervention, defined as a “fragment”, with a univocal, sequential, and speaking code (“unique fragment ID”). This allowed researchers to explore the data reliably. The DB consisted of 2,263 fragments, including the moderator's interventions; 713 fragments were related to the four principal OFGs, and 1,550 fragments were related to the nine teacher OFGs. At the same time, the research team recorded comments made using Zoom's online chat facility.

As regards the lexicometric measures of the various focus groups, they demonstrated that their development is quite similar in terms of words. The lexicometric data relating to the various focus groups showed that, in general, the focus groups had a similar development in terms of words spoken by the participants and by the moderator (total number of words, tokens, are in fact the total occurrences of a focus group). However, as was noted in the debriefing sessions, in some regions, there was a greater number of words (e.g., South school principals, Campania teachers, Emilia Romagna teachers, respectively 15,366, 16,007, 15,289 tokens), and the number of words was also relatively independent of the time duration (as indicated by the token/minute ratio) (respectively 19.21; 18.59; 17.77).

According to one of the main lexicometric measures of the size of the corpus, the whole database consisted of 192,397 tokens and 9,658 types. It is a medium-sized corpus that is positioned almost at the limit of a large corpus^18^ (Giuliano, 2004, p. 64–65).

#### 4.2.5. Focus group data are qualitative

Focus groups are designed to collect more in-depth qualitative data on the participants' experiences through group interaction on a topic determined by the researcher (Morgan, 1997, p. 6). These participants' attitudes and points of view are gained through predetermined stimuli submitted by the moderator. The research team defined stimuli wording that was adjusted to an online setting; the stimuli were shown on slides and after that put in the chat. In this way, participants could read the stimuli in the chat and engage in debate, without distraction. The following analysis of the OFGs was carried out according to a phenomenological-interpretative perspective (Moustakas, 1994; Merriam, 1998), thus proceeding by means of textual content analysis (Losito, 2002; Mayring, 2014). An abductive approach was developed for computer-aided text coding, which combined a bottom-up and inductive approach, emerging from the exploration of the empirical base (textual corpus), with a deductive and top-down approach, based on interpretive categories of the project reference framework (Fereday and Muir-Cochrane, 2006; Adu, 2019).

#### 4.2.6. Focus groups constitute a focused discussion

An OFG is a computer-mediated communication event in which a select group of individuals participates in a moderated, structured, online discussion to explore a particular topic for the purpose of research (Peacock et al., 2009). According to Turney and Pocknee (2005), the OFG, much like the face-to-face focus group, being very natural, is a focused approach, because participants can engage in debate on specific topics by invitation.

The research team defined four stimuli based on the hypothesis of the *VfS* case study. Stimuli were used to guide/ensure comparable conversation through all 13 OFGs. In our study, the moderator's role was most important in setting the context, guiding the discussion, and engaging participants in an interactive conversation.

## 5. Conclusion

COVID-19 prompted discussion on conducting qualitative research in the pandemic era, and there exists a growing body of literature on the practical, technical, and ethical protocols of virtual research modalities, such as video interviews or OFGs. Reflections have included considerations on participant recruitment, ethical consideration about data privacy, and participant digital literacy (Nobrega et al., 2021; Keen et al., 2022; Monaco, 2022). OFGs can become a methodological tool to conduct high-quality and rigorous qualitative research in a context of crisis and beyond, involving a large audience of people by dematerializing participation and thus encouraging green investments.

In this study, we drafted an online focus group procedure with teachers and school principals and used it to process the evaluation of the *VfS* Project. Online modality adjustment of focus groups affected many aspects of the study: (1) designing research stimuli, (2) choosing the technological tools, (3) recruiting participants, (4) scheduling the workflow, and (5) scheduling data analysis. This OFG protocol made it possible to keep methodological and procedural choices under control. It can be used as a working tool for creating upcoming versions of focus groups to assist in the evaluation of what has been achieved up to a certain point and to proceed forward by summarizing strengths and weaknesses. The protocol enabled scheduled phase development and careful definition of the facilitation roles, which is very important in making an OFG successful. The most important elements that made the work positive were (a) the presence of several figures with different roles and complementary functions and (b) the management of the four researchers, which made the meetings extremely fluid, especially concerning the first running-in phases. Using one moderator and two co-hosts was effective for allowing debate, managing participant interactions, directing stimuli presentation, steering other facilitation tools, controlling audio recording, and timekeeping. Assigning a fourth researcher as a co-host observer was useful for collecting data about the facilitation process, participation, and the non-verbal language of the participants. A strength of the protocol was that 13 OFGs were conducted, which allowed the staff to make iterative improvements with each session.

The audio-visual tools facilitated clearer recordings, and the technology-assisted transcription tools captured precise dialog. Participants' webcams were always on; the souvenir photo with the keywords offered a less formal and more convivial final moment.

The Zoom platform (with a Business license) was confirmed as an excellent choice, both in scheduling and managing online focus groups (meeting mode).

The time available was not always sufficient to answer calmly in a well-articulated way and with diversified rhythms. The scheduled time of 1.5 h proved to be rather tight and was almost always exceeded: on average, the duration of the focus groups was approximately 2 h or a little less. The staff identified some areas for future improvement, including fewer stimuli and adjusting participant numbers to relax the pace of discussion. A further limitation of the generalization of the OFG findings is that the participants represent a specific professional sector (teachers and principals) who already had experience using videoconferencing for teaching during the COVID-19 pandemic. It's likely that the protocol could be enhanced for less experienced participants with videoconferencing technology.

In the context of this study, the online focus group as a formal research method met the key criteria of traditional focus group methods, as outlined by Krueger and Casey (2014). Turney and Pocknee (2005, p. 39) “recommend using [virtual] online focus groups more regularly and evaluating their usage in a variety of contexts to confirm these findings”. To increase the validity and reliability of the findings, in our opinion, it is necessary: (1) to pay ever greater attention to the choice of cases, also considering negative (or absent) and deviated cases; (2) to encourage researchers to clearly document their protocol, procedures, setting, equipment, data collection, and analysis methods for others to learn from Tran et al. (2021); (3) to develop innovative methods that enhance participant interaction and build social context through strong moderator leadership skills; and (4) to include effectiveness measures in the protocol.

Further evidence of validity, suggestions, adaptations, and limitations of use for online focus groups can derive from quasi-experimental approaches that include a control group using traditional face-to-face modalities. These research designs could also highlight any differences between online and traditional focus groups in terms of analysis depth, variety of ideas, expressions, and the dynamics of participation.

## Data availability statement

Raw data supporting the conclusion of this article will be made available by the authors, as well as further reflection with other researchers.

## Ethics statement

Ethical review and approval was not required for the study on human participants in accordance with the local legislation and institutional requirements. The participants provided their written informed consent to participate in this study.

## Author contributions

DP was responsible for discussion, validity and reliability, OFG evaluation effectiveness, and conclusion section with LG. MP was responsible for the step-by-step OFG protocol, OFG management, and ethical consideration sections. GP was responsible for the materials and equipment, the videoconferencing system, the word clouds, souvenir photo with the keywords, data analysis protocol, transcription and database building, and textual content coding and analysis sections. LG was responsible for the introduction, online focus group, study design, OFG evaluation form, and conclusion sections with DP. All authors contributed to the study design and approved the final version of the manuscript for submission.

## Footnotes

^1^Using a chat external to the videoconferencing environment (such as a WhatsApp or Telegram group) is advisable not only if the environment has an exclusively private one-to-one chat (as in our case) but also to avoid sending a message to all participants or a private message to one of them due to trivial errors of distraction.

^2^Refer, in particular, to the second reference for a review of the videoconferencing services available and a guide on what services might best suit a project's needs.

^3^Bitly (https://app.bitly.com).

^4^Chat was little used, presumably because, with up to 12-13 participants, in a time frame of approximately 2 h, one can intervene quite easily when one wishes. Chat, on the other hand, proved to be a useful emergency tool for the inclusion in the discussion of a few participants with audio-visual connection difficulties due to bandwidth problems with their Internet connection.

^5^Purposes for the programmatic use of the instant survey in OFGs could include: gathering information on the participants; breaking the ice at the beginning of the meeting (when there is often a bit of embarrassment/awkwardness in intervening directly); and briefly collecting the group's differences of opinion on a stimulus or its particular aspect (proposing alternatives or reporting the main opinions that have emerged on the issue) to encourage/foster discussion, to probe, or to synthesize.

^6^A word cloud – as defined in the Collins English Dictionary – is “a visual representation of the words used in a particular piece of text, with the size of each word indicating its relative frequency”.

^7^Web Speech (https://www.google.com/intl/it/chrome/demos/speech.html).

^8^Dictation.io (https://dictation.io/speech).

^9^Some of the best free solutions include: https://www.wordclouds.com, https://www.visual-thesaurus.com/wordcloud.php, https://worditout.com, https://wordart.com, https://wordsift.org, https://monkeylearn.com/word-cloud, https://www.jasondavies.com/wordcloud.

^10^Voyant Tools (https://voyant-tools.org).

^11^The stop-word list, previously prepared in txt format, contained the so-called empty words (articles, prepositions, conjunctions, demonstrative adjectives, etc.) and participants' names and surnames.

^12^Cabolo (https://www.cabolo.com), developed by Cedat85 (https://www.cedat85.com/it/product/cabolo), is an automatic transcription system based on artificial intelligence technology capable of correctly recognizing a certain number of speakers. The service was purchased in its web version after a comparative evaluation of several similar products.

^13^The progress of the transcription and revision work, carried out by two researchers, was gradually recorded in an MS Excel file and shared with the staff.

^14^The other variables are data on the training course attended (macro-region, methodological learning approach); data on the meetings (the date and a code identifying their type, primarily teachers' or school principals' OFGs); information on the speakers' affiliations (school denomination, technographic code, region, province); a series of data on the participants themselves (name, surname, gender, age, school role, school grade, length of service, seniority in their school, attendance of other training courses on evaluation and their duration).

^15^Text data mining techniques could be used on a later stage in a confirmatory key to verify and support the evidence to be obtained from the qualitative analysis and as an in-depth study of specific and circumscribed aspects.

^16^If contiguous units of analysis attributable to the same code occur within the same fragment, the code must be assigned without interruption, extending the unit of analysis; conversely, if, within the same fragment, there are two or more non-contiguous units of analysis attributable to the same code, the code must be assigned separately; if the same unit of analysis presents different clauses/propositions that can be clearly traced back to different codes, then the coder will proceed with the minute coding of such clause/proposition, breaking down the unit of analysis; if the same unit of analysis presents two or more topics that have to be traced back to different codes but cannot be clearly attributed to distinct clauses/propositions (i.e., it is not possible to apply rule c), the coder will overlap such codes on the same unit of analysis.

^17^QDA Miner, a qualitative data analysis software, developed by Provalis Research (https://provalisresearch.com/products/qualitative-data-analysis-software).

^18^A medium-sized corpus is between 50,000–100,000 occurrences; a corpus greater than 200,000 occurrences is large-sized.
